# A compound reflects the level of homocysteine based on Rhodamine B and its ability to respond to homocysteine in the plasma of diabetic patients

**DOI:** 10.1002/jcla.23202

**Published:** 2020-01-29

**Authors:** Weiyu Zhao, Han Cheng, Yu Zhu

**Affiliations:** ^1^ Tianjin Medical College Tianjin China; ^2^ Department of Clinical Laboratory Tianjin Huanhu Hospital Tianjin China

**Keywords:** diabetic patients, homocysteine, rhodamine B

## Abstract

**Background:**

The level of homocysteine (Hcy) is significantly elevated in the plasma of patients with diabetes. The increased plasma Hcy level is positively correlated with the severity of the disease and is one of the important causes of diabetic complications.

**Methods:**

We designed and synthesized a compound could reflect the level of Hcy based on rhodamine B, and the structure was verified by 1H‐NMR and EI‐HRMS. Then, the linearity, repeatability, selectivity, and cellar toxicity, the effects of the fluid viscosity and pH of compound on Hcy were measured; meanwhile, the response of Hcy level in the plasma of diabetic patients was detected.

**Results:**

This is a novel compound that has never been reported. The compound showed a satisfactory linear range and repeatability at the viscosity and pH of physiological fluid. In addition, the compound was capable of evading the interference from other amino acids and metal ions, and it exhibited high selectivity toward Hcy.

**Conclusion:**

The compound showed increased responsiveness to plasma Hcy in patients with diabetes in comparison with healthy individuals and effectively reflected plasma Hcy levels in healthy individuals and diabetic patients. Therefore, the compound is expected to be used in the diagnosis of diabetes mellitus.

## 1 INTRODUCTION

Diabetes mellitus is a common disease that endangers human health, and the age of patients with diabetes has shown a trend of getting younger.^1^ Diabetes causes a variety of complications such as microvascular diseases, seriously affects the quality of life of the patients, and imposes enormous mental and economic burdens on the patients and their families.^2^, ^3^, ^4^ Early prevention is one of the important measures to alleviate the harm of diabetes. Many studies have examined the development of effective detection technology for the early screening and diagnosis of diabetes.

Homocysteine (Hcy) is a sulfur‐containing amino acid and an important intermediate produced in the metabolism of methionine and cysteine. Under normal conditions, Hcy can be catabolized in the body, which maintains a low serum Hcy concentration.^5^, ^6^ However, in daily life, primary and secondary causes may affect the metabolism of serum Hcy, resulting in the accumulation of Hcy in the blood and an increase in the serum Hcy concentration.^7^, ^8^ Clinical and epidemiological data have confirmed that the Hcy level is higher in patients with diabetes compared with healthy individuals and positively correlated with the severity of the disease.^9^, ^10^ Homocysteine has a toxic effect on vascular endothelial cells and causes vascular endothelial dysfunction/damage as well as lipid peroxidation.^11^ An increased Hcy level is one of the main causes of diabetic complications.^12^ Therefore, the monitoring of Hcy is of great significance for the early screening and diagnosis of diabetes.

Our research group designed and synthesized a pyrrole‐type thiol compound and a nucleophilic addition reaction would occur specifically between the compound and the amino and sulfhydryl groups in Hcy. The spatial structure of the conjugate would change, resulting in an altered fluorescence intensity in the system. The compound can effectively reflect the Hcy level in vitro.

## 2 METHODS

### 2.1 Compound synthesis

**Figure 1:**
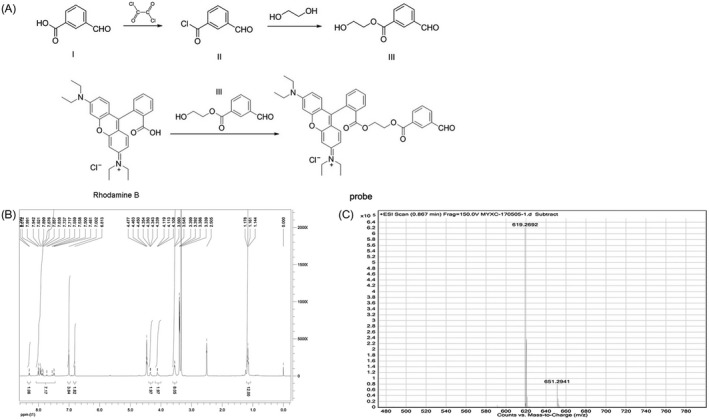
The docking model and synthesis pathway of compound. A, The synthesis pathway of compound; B, The ^1^HNMR result of compound; C, The EI‐HRMS result of compound

Compound I (4‐formylbenzoic acid, 15.0 g, 0.1 moL) was dissolved in 100 mL of dichloromethane, and five drops of dimethylformamide (DMF) were added as a catalyst. The solution was cooled to approximately 0°C in an ice bath. After the dropwise addition of oxalyl chloride (19.05 g, 0.15 moL), the reaction was allowed to continue for 2 hours at room temperature (RT). The reaction system was subjected to rotary evaporation to remove the solvent and excess oxalyl chloride. The resulting compound II (4‐formylbenzoyl chloride) was used directly in the next step without purification. Specifically, 50 mL of ethylene glycol was placed in a reaction flask and cooled to approximately 0°C in an ice bath. Compound II obtained in the above steps was gradually added to ethylene glycol. Again, the reaction was allowed to continue for 2 hours at RT. The reaction system was then subjected to rotary evaporation to remove excess ethylene glycol. The crude product was purified by silica gel column chromatography using petroleum ether/ethyl acetate (ratio 2:1) as the elution solvent, which gave rise to a colorless waxy solid. The solid was compound III (2‐hydroxyethyl‐4‐formylbenzoate, 17.2 g, 88.66%). Rhodamine B (2.39 g, 5 mmoL) was dissolved in 30 mL of dichloromethane, which was sequentially mixed with compound III (1.16 g, 6 mmol/L), EDC·HCl (0.96 g, 5 mmol/L) and 4‐DMAP (0.12 g, 1 mmol/L). The reaction was carried out at RT for approximately 24 hours (traced with thin layer chromatography (TLC), dichloromethane/methanol = 10:1). The reaction system was subjected to rotary evaporation to remove the dichloromethane solvent. After repeated purification by silica gel column chromatography (eluent: dichloromethane/ methanol = 10:1), the dark red powder was obtained as Figure 1B. The compound structure was analyzed by ^1^H‐NMR and EI‐HRMS (Agilent Technologies, Inc).

### 2.2 Determination of maximum emission wave length and linearity of the fluorescence measurement of Hcy

The working solution of the compound (10 μmol/L) was prepared with phosphate‐buffered saline (PBS). A black 96‐well microplate was filled with PBS and 20 μmol/L of Hcy (100 μL per well). After incubation at 37°C for 10 minute, the fluorescence intensity with different emission wavelength was measured (excitation wavelength 280 nm) by microplate reader (Thermo Fisher Scientific Inc).

A black 96‐well microplate was filled with 20, 40, 60, 80, and 100 μmol/L of Hcy (100 μL per well). After incubation at 37°C for 10 minutes, the fluorescence intensity of each well was measured (excitation wavelength, 280 nm; emission wavelength, 590 nm) by microplate reader, and the linearity was calculated.

### 2.3 Determination of the repeatability of the fluorescence measurement of Hcy

The working solution of the compound (10 μmol/L) was prepared with PBS. A black 96‐well microplate was filled with 20, 40, 60, 80, and 100 μmol/L of Hcy (100 μL per well). After incubation at 37°C for 10 minutes, the fluorescence intensity of each well was measured with an excitation wavelength of 280 nm and emission wavelength of 590 nm by microplate reader. The measurement was repeated 6 times, and the repeatability was calculated.

### 2.4 The effects of the fluid viscosity and pH on compound responsiveness

The working solution of the compound (10 μmol/L) was prepared with PBS. To analyze the effect of the fluid viscosity on compound responsiveness, a 96‐well black microplate was filled with 100 μL of 20 μmol/L Hcy. After the addition of 100 μL of the compound, the plate was incubated at 37°C for 10 minutes, and the fluorescence intensity was measured by microplate reader (excitation wavelength, 280 nm; emission wavelength, 590 nm). To examine the effect of the pH on compound responsiveness, buffers with pH values of 6.5‐8.5 were prepared. The buffers, together with 10 μmol/L compound, were added to 20 μmol/L Hcy and incubated at 37°C for 30 minutes. The fluorescence intensity was measured by microplate reader (excitation wavelength, 280 nm; emission wavelength, 590 nm).

### 2.5 Determination of the compound selectivity

Considering the complexity of the intracellular environment, the interfering substances selected for the assay were the main amino acids and small molecules present in organisms. The interfering substances included other amino acids (cysteine (Gys), Hcy, N‐acetylcysteine (NAC), glutathione (GSH); glutamine (Gln); glutamic acid (Glu); and tyrosine (Tyr) (1 mmol/L each). The working solution of the compound (10 μmol/L) was prepared with PBS. The above substances were added to a 96‐well black microplate, followed by the addition of 100 μL of the compound. After incubation at 37°C for 10 minutes, the fluorescence intensity of each well was measured (excitation wavelength, 280 nm; emission wavelength, 590 nm) by microplate reader, and the selectivity of the compound to the interfering substances was analyzed.

### 2.6 Detection of Hcy in the serum of diabetic patients using the compound

In this study, 50 type 2 diabetes mellitus patients (diabetic group, age 56.9 ± 8.6) with fasting plasma glucose ≥ 7.0 mmol/L and diagnosed in Tianjin Huanhu Hospital from January 2018 to November 2018 were enrolled in this study according to American Diabetes Association Standards of medical care in diabetes.^13^ Fifty healthy volunteer was as normal group (Norm group, age 54.0 ± 6.1). All participants in the experiments were matched for age and gender. This study was approved by the medical ethics committee of Tianjin Huanhu Hospital on 2017 according to the Declaration of Helsinki, and patients provided informed consent before the experiments (ethics approval number is 2017‐5). The exclusion criteria: (a) functional insufficiency of heart, lung, liver, and kidney; (b) cerebral infarction, hyperthyroidism, hypothyroidism, and other systemic diseases; (c) infection, ketoacidosis, hyperosmolarity, severe hypoglycemic coma, or other urgent complication; (d) recently taken medication that is likely to affect cognitive function.

Venous blood (5 mL) were collected from patients and healthy volunteer and centrifuged at 300 g for 10 minutes with room temperature. The content of Hcy in serum was measured by biochemistry analyzer (Beckman Coulter). Dithiothreitol (1 mol/L, 5 μL) (Sigma‐Aldrich Ltd.) was added in to serum above‐mentioned (95 μL) incubation at 37°C for 30 minutes, and then, the compound (10 μmol/L) was added incubation at 37°C for 30 minutes, the fluorescence intensity was measured at an excitation wavelength of 280 nm and an emission wavelength of 590 nm by microplate reader (Thermo Fisher Scientific Inc).

### 2.7 Statistical analysis

Data were represented by mean ± SD and analyzed by SPSS 11.0 software. The one‐way ANOVA analysis and Tukey's post hoc test was used for general measurement data. Pearson's correlation coefficient used to correlative analysis. *P* < .05 was defined as a significant difference.

## 3 RESULTS

### 3.1 Compound structure analysis

The following results were obtained by mass spectrometry and nuclear magnetic spectroscopy (Figure 1C,D): ^1^HNMR (400 MHz, DMSO‐D6) δ 10.14 (s, 1H, −CHO), 8.30 (d, J = 6.8 Hz, 1H), 8.01‐7.84 (m, 5H), 7.74‐7.50 (m, 2H), 7.00 (m, 4H), 6.81(s, 2H), 4.35 (t, J = 1.6 Hz, 2H), 4.11 (t, J = 2.4 Hz, 2H), 3.55 (q, J = 6 Hz, 8H), 1.16 (t, J = 6.8 Hz, 12H); EI‐HRMS (C38H39N2O6+). Molecular weight: 619.27. Thus, the obtained compound was identified as the target compound.

### 3.2 The linearity of the compound in the detection of Hcy

**Figure 2:**
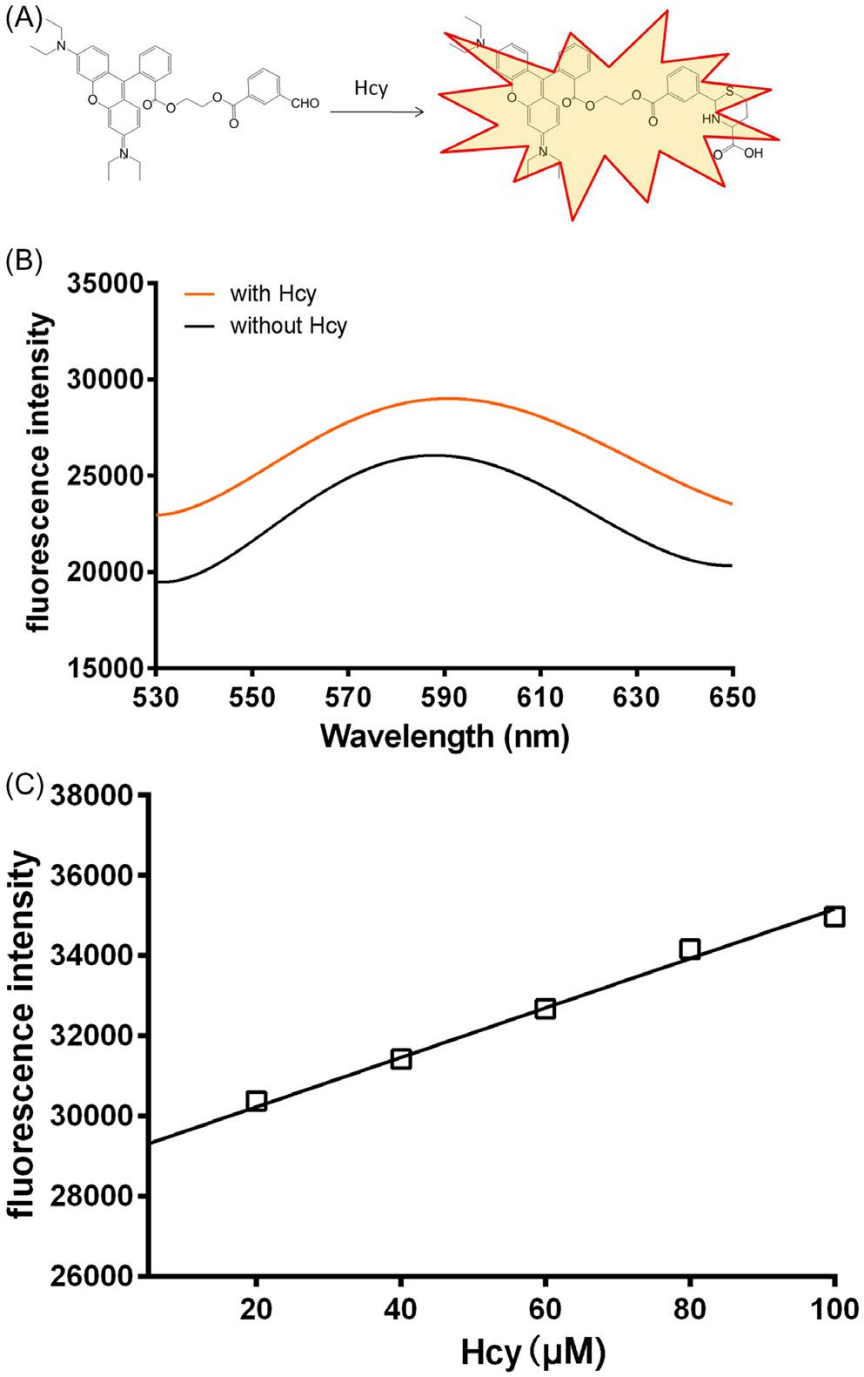
The linearity of the detection of Hcy by the compound. A, The conjugated model of compound and Hcy; B, The graph showed that the 590 nm was the most appropriate excitation wave length; C, The graph showed that there was a well linearity of the detection of Hcy by the compound in the concentration range of 0‐100 μmol/L. *Y* = 61.64*X* + 29 002, *r* = .9953

The model of compound was conjugated with Hcy as shown in Figure 2A, and the most appropriate excitation wave length was 590 nm as shown in Figure 2B. The compound was allowed to react with Hcy. A linear plot of the reaction of the compound with Hcy was established after subtracting the autofluorescence of the compound (Figure 2C, *Y* = 61.64*X* + 29 002, *r* = .9953). The above results showed that the compound exhibited good linearity for Hcy.

### 3.3 Examination of the repeatability of the fluorescence measurement of Hcy

A repeatability experiment was performed on the fluorescence intensity after the compound reacted with Hcy. It was confirmed that the results of Hcy detection obtained using the compound were highly stable and reliable (Table 1).

**Table 1:** The repeatability of the fluorescence measurement

Hcy (µmol/L)	Fluorescence intensity					Mean	SD	CV (%)
0	28 895	28 901	28 865	28 845	28 896	28 880.4	24.35	0.08
20	30 276	30 428	30 371	30 442	30 338	30 371.0	67.83	0.22
40	31 447	31 358	31 458	31 536	31 320	31 423.8	85.73	0.27
60	32 619	32 627	32 843	32 853	32 447	32 677.8	171.24	0.52
80	34 017	34 125	34 291	34 166	34 242	34 168.2	106.37	0.31
100	34 957	35 023	34 987	34 942	34 994	34 980.6	31.88	0.09

### 3.4 The effects of the fluid viscosity and pH on compound responsiveness

**Figure 3:**
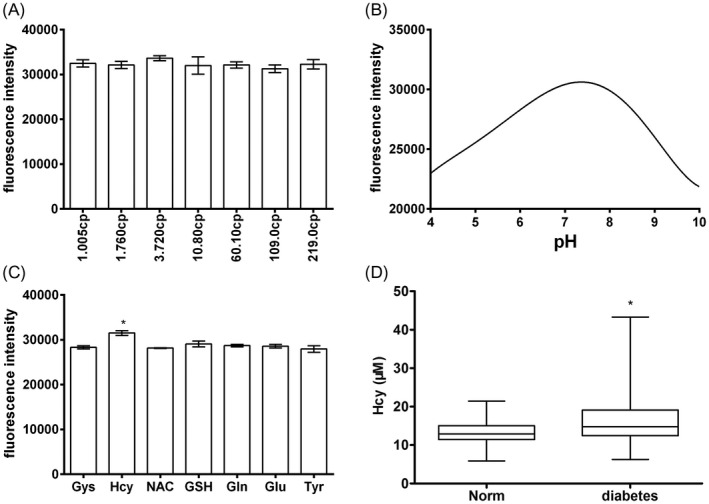
The effect of fluid viscosity, pH on compound and its selectivity and cellar toxicity. A, The graph showed that there was a stable response to Hcy at different viscosities (glycerol/water, 1.005‐219 cp). B, The graph showed that there was a stable responsiveness to Hcy at pH = 7. C, The graph showed that there was a significant increased fluorescence intensity of the compound in Hcy compared with other biological analyte. ^*^ *P* < .05 compared with other biological analyte. D, The graph showed that there was a significant increased level of Hcy in diabetic patients group compared with Normal group by compound. ^*^ *P* < .05 compared with normal group

The compound showed good responsiveness to Hcy at different viscosities (glycerol/water, 1.005‐219 cp), which renders it possible to avoid the potential impact of serum viscosity changes caused by physiological function changes or disease on compound responsiveness (Figure 3A). In addition, the compound showed good responsiveness to Hcy at a pH of approximately 7, which may avoid the impact of pH changes caused by physiological function changes or disease on compound responsiveness (Figure 3B).

### 3.5 Compound selectivity and cellar toxicity

Incubation of the compound with the interfering substances described above, including cysteine, Hcy, N‐acetylcysteine, glutathione, glutamine, glutamic acid, and tyrosine, did not affect the detection of Hcy (Figure 3C).

### 3.6 The response of the compound to Hcy levels in the plasma of diabetic patients

The compound could well detect the level of Hcy in serum of healthy people and type 2 diabetes mellitus patients, and we found that the compound could distinguish the different level of above groups (Figure 3D).

## 4 DISCUSSION

Hcy is a sulfur‐containing amino acid that cannot be synthesized in vivo. Homocysteine can only be derived from the catabolism of methionine.^14^ Hyperhomocysteinemia is more pronounced in diabetic patients with concurrent renal, retinal or cardiovascular complications, diabetes‐related heart disease, and stroke.^15^, ^16^, ^17^, ^18^ Homocysteine has become an important diagnostic molecule for diabetes, and it is of great significance to develop Hcy compounds with potential clinical application value.

Rhodamine B was used as the mother nucleus in this study. The compound was identified as an unreported new compound. Examination of the fluorescence intensity revealed that a nucleophilic addition reaction had occurred specifically between the compound and the amino and sulfhydryl groups in Hcy. The spatial structure of the conjugate was changed, resulting in the enhancement of fluorescence intensity in the system. Due to the structural similarity among the small‐molecule sulfhydryl compounds, direct detection of various sulfhydryl compounds (such as cysteine and glutathione) with small‐molecule fluorescent compounds has rarely been reported. Our compound selectively detected Hcy; meanwhile, it did not respond to cysteine and other amino acids. Such good selectivity may be related to the binding of the compound to specific residues in Hcy, which needs to be further verified in future studies.

The plasma level of Hcy is below 100 μmol/L in most healthy individuals and patients. Linear analysis demonstrated that the compound exhibited good linearity for Hcy in the concentration range of 0‐100 μmol/L and effectively reflected the plasma Hcy levels in most healthy individuals and patients. The present study found that compound generated significantly different signals in the plasma of healthy individuals and patients with diabetes, which were correlated with the Hcy results obtained in the above two groups using a biochemical analyzer. The findings indicate that compound can be used as a compound for Hcy detection.

The compound exhibited good water solubility, sensitivity, and selectivity. It not only showed high linearity and repeatability for Hcy solution but also effectively reflected the Hcy level in the serum. Examination of clinical specimens with the compound showed that the fluorescence signal of the compound was significantly higher in diabetic patients compared with the healthy control group, indicating that the compound has potential clinical application value. We plan to further increase the number of specimens in the future and conduct comparisons with the existing detection methods in hope of developing a novel type of Hcy‐detecting compound.
